# Thickness dependence of structural and superconducting properties of Co-doped BaFe_2_As_2_ coated conductors

**DOI:** 10.1016/j.isci.2021.102922

**Published:** 2021-07-30

**Authors:** Zhongtang Xu, Chiheng Dong, Chuanbing Cai, Pusheng Yuan, Yanwei Ma

**Affiliations:** 1 Key Laboratory of Applied Superconductivity, Institute of Electrical Engineering, Chinese Academy of Sciences, Beijing 100190, China; 2 University of Chinese Academy of Sciences, Beijing 100049, China; 3 Research Center for Superconductors and Applied Technologies, Shanghai University, Shanghai 200444, China; 4 State Key Laboratory of Functional Materials for Informatics, Shanghai Institute of Microsystem and Information Technology, Chinese Academy of Sciences, 865 Changning Road, Shanghai 200050, China

**Keywords:** Superconductivity, Condensed matter properties, Energy materials, Solid state physics

## Abstract

High-quality Co-doped BaFe_2_As_2_ thin films with thickness up to 2 μm were realized on flexible metal tapes with LaMnO_3_ as buffer layers fabricated by an ion beam-assisted deposition technique. Structural analysis indicates that increasing thickness does not compromise the film crystallinity, except for a small amount of impurities. Two types of thickness dependence of critical current density (*J*_c_) were found: one is almost thickness independent in the range of 0.6–1.5 μm and the other is highly thickness dependent. In addition, the maximum value for crucial current *I*_c_ at 9 T and 4.2 K is about 55 A/12 mm-W for the 1.5-μm-thick film. Anisotropic Ginzburg–Landau scaling demonstrates that dominant pinning centers develop from correlated to uncorrelated with increasing film thickness. The further theoretical analysis shows that with film thickness increasing the pinning mechanism evolves progressively from a *δl* pinning to the *δT*_*c*_ pinning mechanism.

## Highlights

• Thickness effect was investigated in Co-doped Ba122 coated conductors up to 2 μm
• A δ*l* pinning to the δ*T*_c_ pinning mechanism with film thickness increasing was observed
• Maximum *I*_c_ at 9 T and 4.2 K is improved up to 55 A/12 mm-W for a 1.5-μm-thick film

## Graphical abstract

**Figure:**
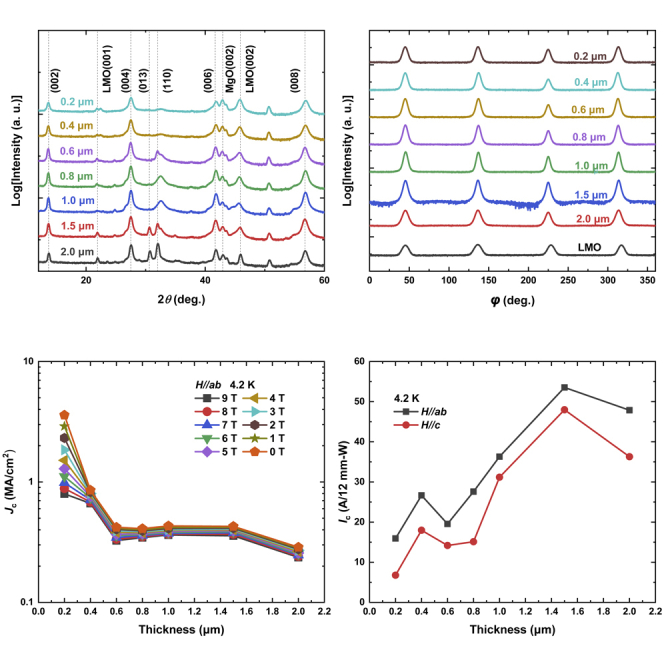

## Introduction

Iron-based superconductors (IBSs), exhibiting high upper critical fields (*H*_c2_), larger critical grain boundary angle (*θ*_c_), small anisotropies (*γ*), and robust superconductivity to disorder, demonstrate an excellent potential for high-field applications (Hosono et al., 2018; Sakoda et al., 2018; Iida et al., 2020). Coated conductor (CC) templates, which have been developed with tremendous success in cuprate-based superconductors, are also favorable for growth of IBS epitaxial films, such as Ba122 (Ba(Fe_1-x_Co_x_)_2_As_2_ and BaFe_2_(As_1-x_P_x_)_2_) (Iida et al., 2011b, 2017; Hiramatsu et al., 2017; Sato et al., 2016; Trommler et al., 2012a, 2012b; Katase et al., 2011; Xu et al., 2018) and 11 thin films (FeSe_x_Te_1−x_) (Sylva et al., 2019; Nabeshima et al., 2017; Demura et al., 2016; Si et al., 2011, 2013; Yuan et al., 2015). In particular, the P-doped Ba122 CCs on the ion beam-assisted deposition (IBAD)-MgO buffered metal tapes show values of critical current densities (*J*_c_) over 0.1 MA/cm^2^ at 15 T (Iida et al., 2017), indicating their excellent potential for high-field applications. However, by now, the thickness of the superconducting layer for IBS CCs is only a few hundred nanometers, resulting in a low critical current (*I*_c_) of such tapes. Increasing the thickness of the IBS coating is of great importance for improving the current-carrying capacity of IBS CCs. Nonetheless, it is important to note that increasing the film thickness can lead to adverse effects on the superconducting performance, i.e. for Co-doped Ba122 thin films on (La, Sr)(Al, Ta)O_3_ (LSAT) (00*l*) single crystal substrates, with increasing film thickness to 1080 nm, weakening of the in-plane and out-of-plane texture and decreasing of *J*_c_ have been observed (Katase et al., 2012). However, few studies have been performed on the thickness dependence of superconducting properties in IBS CCs. With extensive research on the thickness effect of cuprate CCs, it has been known that *J*_c_ decreases rapidly as the thickness of cuprate coating layer increases, independent of deposition method, and additionally crystalline orientation varies as thickness increases and coating layer morphology also becomes rougher (Foltyn et al., 1999; Kang et al., 2002). Considering that cuprate and IBSs share a high similarity, such as a layered structure, very high upper critical fields and transition temperatures, a doping phase diagram, thickness effect may also play an important role in determining the superconducting properties of IBS CCs.

In this study, we focus on the effect of film thickness on the superconducting properties of BaFe_1.84_Co_0.16_As_2_ (Ba122:Co) thin films on IBAD metal tapes. The thickness effects on the structure and transport properties were investigated. Evolution of the pinning mechanism with film thickness was analyzed by the *δl* and *δT*_*c*_ pinning model.

## Results

### Structure and morphology characterization of Ba122:Co CCs

**Figure 1:**
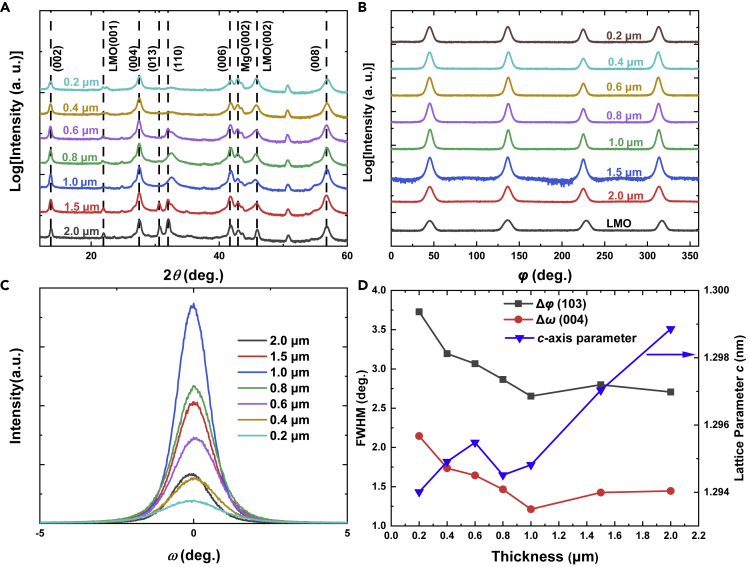
Structure characterization of Ba122:Co CCs (A) Typical out-of-plane XRD patterns of Ba122:Co thin films with different thickness on IBAD-LMO buffered metal tapes. (B) φ scans of the Ba122:Co (103) thin films with different thickness and the LMO (101) diffractions. (C) Evolution of the out-of-plane rocking curve of the (004) diffractions with thickness. (D) Dependence of the FWHM of the φ scan and rocking curve for (103) and (004) diffractions, and the *c*-axis parameters on film thickness.

Figure 1A displays the variation of x-ray diffraction (XRD) patters of Ba122:Co CCs with thickness. Mainly *c*-axis orientation can be observed up to the maximum thickness of 2 μm. However, with increasing thickness, besides the (00*l*) peaks, diffraction peaks coming from other orientations are clearly visible, e.g. (110) peak starts at 0.6 μm and (013) peak at 1.5 μm. This demonstrates that randomly oriented grains occur for thicker films. Similar phenomenon due to thickness effect is also observed in Ba122:Co films on LSAT single crystal substrates (Katase et al., 2012) and on MgO substrates with an Fe buffer layer (Iida et al., 2011a). However, in our case, the appearance of randomly oriented grains results in virtually less degradation of the matrix crystalline quality, as evidenced by the *φ* scan of (103) peak and rocking curves of (004) peak in Figures 1B and 1C. It can be seen from Figure 1B of the *φ* scans around the (103) peak of the films and the (101) peak of the LaMnO_3_ (LMO) buffer layer that each films of different thickness show a clear four-fold symmetry corresponding to the tetragonal structure of the Ba122:Co lattice, and the diffraction peaks of the films are aligned with those from the buffer layer, indicating an in-plane alignment between the films and buffer layer. In addition, the out-of-plane rocking curves of the (004) diffractions of different thickness in Figure 1C also indicate a high texture. The full width at half-maximum (FWHM), Δ*ω*, of the (004) rocking curve and the average FWHM values of the four peaks in the *φ* scan of the (103) diffraction of the thin films, Δ*φ*, are summarized in Figure 1D. It can be seen that both the FWHM values become smaller as thickness increases up to 1μm and eventually show a saturation tendency at thicker films. It is also worth mentioning that the FWHM values of 2.5–3° at thicker films for the (103) diffractions are much less than that of the LMO buffer layers of (101) diffractions (FWHM∼5.7°). The variation of FWHM values with film thickness may suggest a reduced role of the buffer layer LMO on the film. In the initial stage, the structure of the LMO has a dramatic impact on the structure of the films. However, the influence of LMO becomes weakened as film grows thicker. Therefore, both the FWHM values of in-plane and out-of-plane diffractions decrease with increasing film thickness up to 1μm. Gradually, the influence of the LMO would be ended at a certain film thickness (1 μm in current case). Meanwhile, the self-assembly behavior and longer deposition time for thicker films would advance the grain-to-grain alignment, leading to a better texture. On the other hand, as in Figure 1D, it exhibits a little change in the *c*-axis parameter ranging from 0.2 to 1 μm, whereas it monotonically increases with increasing thickness up to 2 μm, and eventually approaches the bulk value of the *c*-axis parameter (Sun et al., 2011), also indicating the reduced role of LMO buffer layer with increasing film thickness.

**Figure 2:**
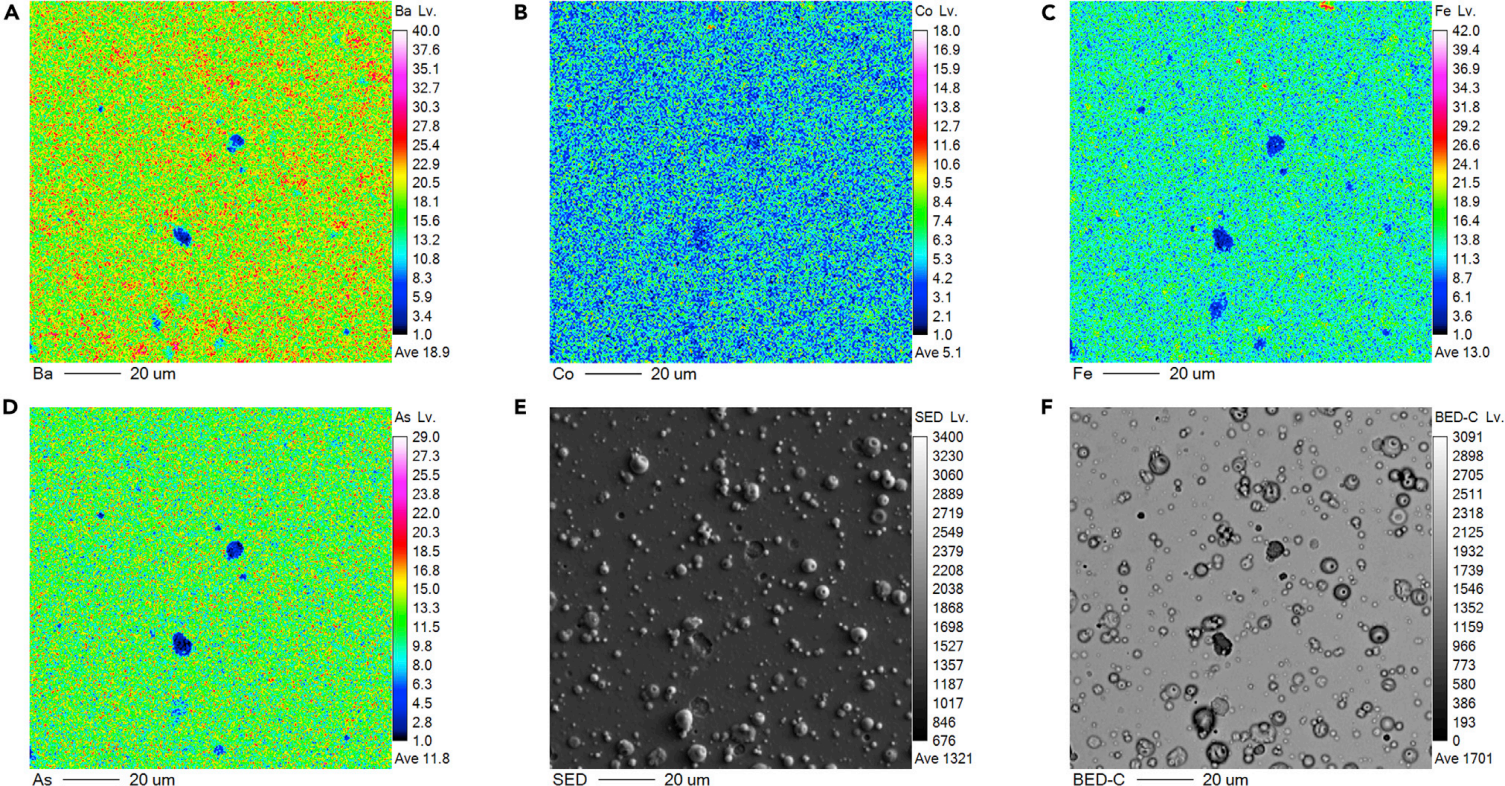
Morphology characterization of Ba122:Co CCs (A–D) Elemental mapping of Ba, Co, Fe, As for the 2μm-thick Ba122:Co thin films, respectively. (E and F) (E) Secondary electron detector and (F) backscattered electron detector images of the Ba122:Co thin films.

Increasing film thickness does not compromise the structure quality as evidenced by the XRD results, whereas the surface topography gets complicated as film thickness increases, as shown in Figure 2A. It can be seen from the scanning electron microscopy image in Figure 2E that droplets and pits can be observed on the 2-μm-thick film. Meanwhile, the composition behaves differently for the droplets and pits, as can been seen from the elemental mapping of the Ba, Co, Fe, As elements and the backscattered electron images, which is sensitive to the chemical composition. Two obviously different contrasts can be seen from the backscattered electron images: a dark contrast in the pit area, and a brighter contrast in the other area, which can be ascribed to the small compositional fluctuations. Meanwhile, a good homogeneity of element distribution except for the droplet and pit areas is confirmed by the elemental mapping, which demonstrates that the element distribution of Ba122:Co phase is homogeneously dispersed. On the other hand, the droplets are slightly Ba-rich, indicative of off-stoichiometry from Ba122:Co target. Such droplets, i.e. particles of larger size typically observed on the surface of films fabricated by PLD technique, such as YBa_2_Cu_3_O_7−δ_ thin films (Huhtinen et al., 1999; Fabbri et al., 2000), which is believed be related to growth parameters, such as the microstructure, surface morphology, and the density of the target. Therefore, further optimization of the growth parameters and target quality is necessary to improve the homogeneity.

### Transport performance

**Figure 3:**
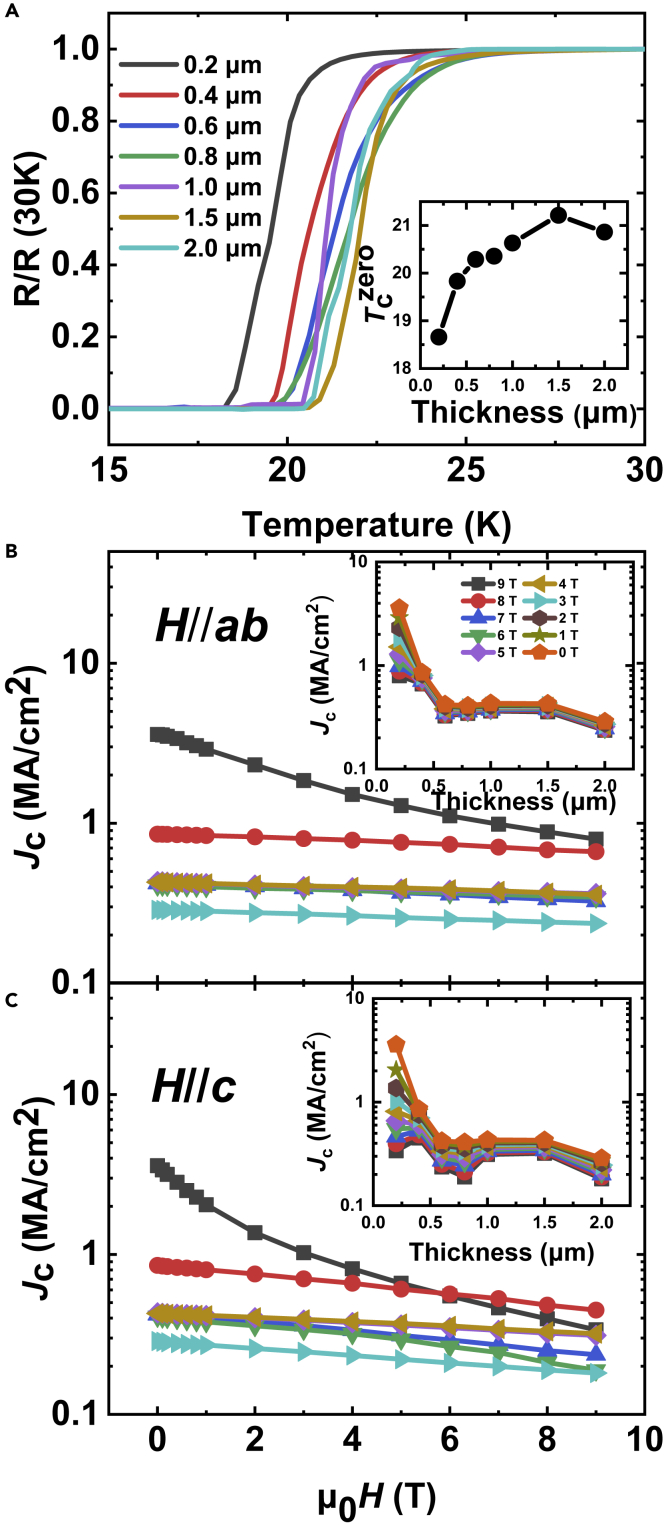
Transport performance (A) Dependence of the normalized resistivity of Ba122:Co films on film thickness in zero-field. The inset shows the *T*_c_^zero^ as a function of film thickness. (B and C) (B) and (C) display the field dependence of the critical current densities *J*_c_s of films at 4.2 K with different thicknesses up to 9T for *H//ab* and *H//c*, respectively. The insets in (B) and (C) give the thickness dependence of *J*_c_s up to 9T for *H//ab* and *H//c*, respectively.

Figure 3A shows the typical temperature dependence of the normalized resistance for Ba122:Co films as a function of thickness in zero field. All films show superconductivity; however, the zero-resistance temperatures *T*_c_^zero^ exhibit film thickness dependence, as shown in the inset of Figure 3A. It can be seen that the *T*_c_^zero^ increases from 18.65 K for 0.2-μm-thick film to 20.86 K for 2 μm thick-film and shows the maximum value of 21.21 K for 1.5-μm-thick film, indicating that effects of thickness on *T*_c_ is weakened with increasing film thickness, and the *T*_c_ of films are closer the bulk *T*_c_ for thicker films. Such a thickness dependence of *T*_c_ may be caused by the weakening role of the LMO buffer layer on Ba122:Co film with thickness, as evidenced by the XRD patterns in Figure 1, with increasing thickness, the *T*_c_ of films recovers more closely to the bulk properties. The field dependence of critical current densities *J*_c_ at 4.2 K for films with thickness of 0.2, 0.4, 0.6, 0.8, 1, 1.5, and 2 μm are shown in Figures 3B and 3C for *H*//*ab* and *H*//*c*, respectively. *J*_c_s for *H//ab* are always higher than those for *H*//*c*, due to the anisotropy of *H*_c2_. Self-field *J*_c_ of 3.5 MA/cm^2^ is recorded for the 0.2-μm-thick film, which is comparable to the similar films on CaF_2_ single crystal substrates (Yuan et al., 2017). *J*_c_s monotonously decrease with increasing field for all films. At 9T for 0.2-μm-thick film, *J*_c_ still retains 0.80 and 0.34 MA/cm^2^ for *H*//*ab* and *H*//*c*, respectively. The insets of Figures 3B and 3C show the thickness dependence of *J*_c_ from zero field to 9 T at 4.2 K for *H*//*ab* and *H*//*c*, respectively. The *J*_c_-*t* (thickness) behavior can be divided into three regions: (I) A monotonic decrease in *J*_c_ as the film thickness increases can be observed up to 0.6-μm-thick film, (II) followed by a range of 0.6–1.5 μm where *J*_c_s are almost thickness independent, (III) and then a quick reduction in *J*_c_ for the 2-μm-thick film. It should be noted that in region (I) the *J*_c_ drops very quickly with thickness, the self-field drops from 3.5 MA/cm^2^ to 0.42 MA/cm^2^, and the *J*_c_ in field from 0.80 (9T, *H*//*ab*, 0.2 μm) and 0.34 MA/cm^2^ (9T, *H*//*c*, 0.6 μm) to 0.33 MA/cm^2^ (9T, *H*//*ab*, 0.6 μm) and 0.23 MA/cm^2^ (9T, *H*//*c*, 0.6 μm), respectively. Meanwhile, such a thickness dependence of *J*_c_ for *H*//*ab* is almost independent of temperature, as shown in Figures S1A and S1C for *J*_c_ at 8 and 12 K, however, a sudden drop at 0.8 μm is observed for *H*//*c* as shown in Figures S1B and S1D.

The thickness dependence of *J*_c_ for our IBS CCs is quite different from that in cuprate superconductors. It has been found that the *J*_c_-*t* behavior in cuprate superconductors can be addressed by collective pinning theory, by which the *J*_c_ reduction with increasing thickness can be well described as *J*_c_ ∼ *t*^−1/2^, followed by a crossover thickness *t*_c_, over which *J*_c_ becomes almost thickness independent (Wang and Wu, 2007; Tran et al., 2013; Gurevich, 2007). It is interesting to note that the thinnest films with the lowest *T*_c_ showed the highest *J*_c_ up to 5 T for *H//c* and 9 T for *H//ab*. A plausible reason is the higher grain boundaries (less than the critical angle of IBSs (9°)) in the thinnest films than thicker films, as evidenced by the largest FWHM values of out-of-plane and in-plane diffractions for the thinnest films (Figure 1D). It has been reported that enhanced *J*_c_ values and significant better pinning performance in P doped Ba122 thin films can be achieved by high density grain boundaries due to poorly aligned IBAD-MgO metal tapes (8°) compared with well-aligned ones (4°), although a lower *T*_c_ and poorer crystallinity were also observed in films with higher grain boundaries (Sato et al., 2016). Furthermore, although the *J*_c_s show an overall progressive reduction with film thickness, the critical current *I*_c_ is enhanced from 16 A/12 mm-width (A/12 mm-W) (4.2 K, *H//ab*, 0.2 μm) up to 55 A/12 mm-W (4.2 K, *H//ab*, 1.5 μm) due to the thickness increase, as shown in Figure S2 for the thickness dependence of *I*_c_ at 4.2 K for *H//ab* and *H//c* at 9T. However, the *I*_c_ values are much less than those of powder-in-tube processed Ba122:K tapes (*I*_c_ = 437 A, at 4.2 K, 9T) (Huang et al., 2017), as well as 1.1-μm-thick YBa_2_Cu_3_O_7−δ_ (YBCO) CCs (*I*_c_∼350 and 1600 A/4 mm-W at 4.2 K and 10 T for *H//c* and *H//ab*, respectively) (Braccini et al., 2010).

## Discussion

**Figure 4:**
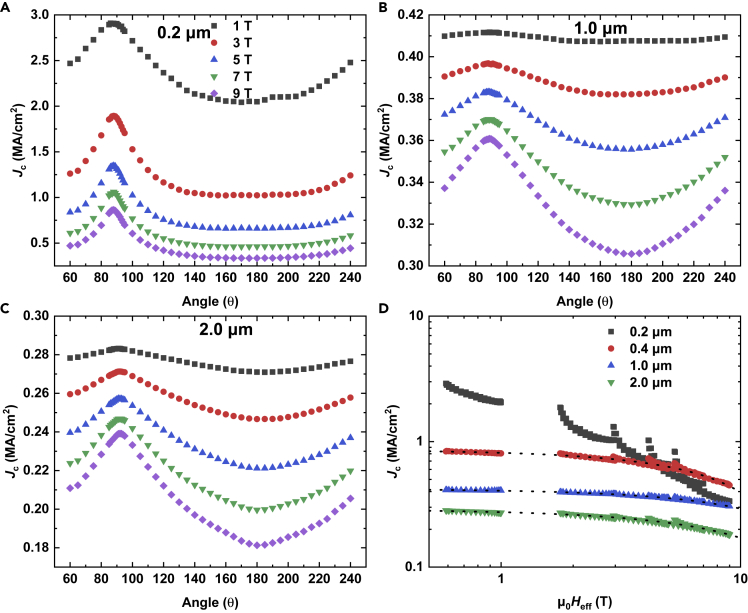
Angular dependence of *J*_c_ and *J*_c_ analysis by anisotropic Ginzburg–Landau scaling (A–C) Angular dependence of *J*_c_ for the Ba122:Co films of 0.2, 1.0 and 2 μm at 4.2 K under fields of 1, 3, 5, 7 and 9 T. (D) Scaling behaviors of *J*_c_(θ) at 4.2 K for Ba122:Co films with thickness of 0.2, 0.4, 1.0 and 2.0μm. The dot lines are guides to the eyes.

To investigate the anisotropy of flux pinning, the *J*_c_ as a function of field orientation, *θ*, under different fields of 1, 3, 5, 7 and 9 T at 4.2 K was measured for 0.2-, 1-, and 2-μm-thick films, as shown in Figures 4A–4C. A broad peak, which becomes more prominent with increasing fields, is always observed for field parallel to the *ab*-plane (*θ* = 90°). Such a peak is typically observed in Ba122 thin films due to their layered structure (Iida et al., 2010b). On the other hand, no peak for field parallel to the *c*-axis (θ = 180°) was observed. According to anisotropic Ginzburg–Landau scaling, if the *J*_c_ anisotropy is caused by the anisotropy of the effective electron mass, then the angular dependence of *J*_c_ should be scaled with an effective field *H*_eff_ = *H*ε(θ), with $\varepsilon \left(\theta \right)=\sqrt{cos^{2}\left(\theta \right)+\gamma^{-2}sin^{2}\left(\theta \right)}$, where *γ* is the effective mass anisotropy (Blatter et al., 1994). This approach can be used to extract the contribution from the random defects. This scaling method was usually used for anisotropic single-band superconductors, however, it also has been applied successfully to describe multiband IBS, such as Ba122:Co thin films (Iida et al., 2010a, 2010b; Hänisch et al., 2014, 2015). The scaling behavior of *J*_c_(*θ*) for 0.2-, 0.4-, 1-, and 2-μm-thick films with respect to *H*_eff_ at 4.2 K is shown in Figure 4D. Owing to the multiband characteristic of IBSs, the anisotropy *γ* is temperature dependent (Iida et al., 2010a, 2010b) and typically has values of 1.4–2.4. In our case, *γ* value of 1.7 at 4.2 K was used for scaling. It can be seen from Figure 4D that the 0.2-μm-thick film cannot be scaled onto a master curve, indicating correlated defects appear. However, with increasing thickness, all data except in the vicinity of field parallel to the *ab* plane collapse onto a single curve, indicating uncorrelated defects dominate in those angular regions. For thicker films, the correlated defects active at high field, as a weak derivation near the *ab*-plane caused by the correlated defects becomes more obvious at high field.

**Figure 5:**
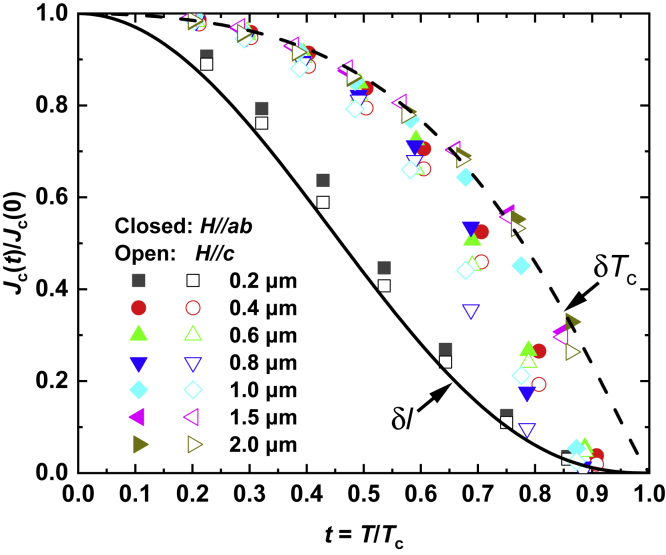
Pinning mechanism of Ba122:Co CCs Reduced temperature dependence of the normalized *J*_c_ at 1 T. The solid and dot lines represent the theoretical prediction curve of δ*l*- and δ*T*_c_-pinning, respectively.

Typically, there are two predominant contributions to the flux pinning in type-II superconductors: *δT*_c_ and *δl* pinning (Blatter et al., 1994). The *δT*_c_ pinning is ascribed to the randomly distributed spatial variation of the Ginzburg parameter *k* caused by the spatial fluctuations in *T*_c_, while *δl* pinning is due to the spatial variation of the mean free path of charge carriers. Griessen *et al.* (Griessen et al., 1994) showed that in the single vortex pinning regime the two pinning mechanism manifest themselves in different reduced temperature dependence of normalized critical current density *J*_c_(*T*)/*J*_c_(0), that is, for *δT*_c_ pinning *J*_c_(*T*)/*J*_c_(0) = [1-*t*^2^]^7/6^[1-*t*^2^]^5/6^, and for *δl* pinning *J*_c_(*T*)/*J*_c_(0) = [1-*t*^2^]^5/2^[1-*t*^2^]^−1/2^, with *t* = *T*/*T*_c_. The normalized *J*_c_ as a function of the reduced temperature *t* = *T*/*T*_c_ for different thickness at 1 T for *H*//*ab* and *H*//*c* is shown in Figure 5, along with the theoretical prediction curves of *δT*_c_ and *δl* pinning. It is clearly seen that the pinning mechanism is closely related to the thickness. The pinning mechanism is very close to the *δl* pinning for the 0.2-μm-thick film, and it is *δT*_c_ pinning for the 1.5- and 2-μm-thick films. In addition, a transformation from the *δl* pinning to the *δT*_c_ pinning with thickness, especially for high temperature, is obviously seen in the intermediate thickness, indicating that *δl* pinning weakens with thickness and both pinning mechanisms coexist. On the other hand, in the intermediate thickness, the main contribution arises from the *δT*_c_ pinning at low temperature and gradually transits into the *δl* pinning in the vicinity of *T*_c_, demonstrating that the dominant pinning mechanism changes with temperature. Such a temperature-dependent pinning mechanism evolution was also reported in YBa_2_Cu_3_O_7-*δ*_ superconductors (Algarni et al., 2021), and Ba122:Co (Shen et al., 2010), and Ba122:K single crystals (Ghorbani et al., 2012). It has been shown that in the similar single crystal Ba(Fe_0.92_Co_0.08_)_2_As_2_ the contribution from the *δT*_c_ pinning is larger than that from the *δl* pinning (Ishida et al., 2017), as in our case for films thicker than 0.2 μm. It has been reported that the *δl* pinning is associated with the intergrain boundaries and intragrain inclusions (Ghorbani et al., 2008). According to the XRD results in Figure 1D, the in-plane misalignment is largest for the 0.2-μm-thick film, therefore, larger intergrain boundaries may develop, causing the film to show a *δl* pinning behavior. With increasing thickness, uncorrelated defects, such as point defects, become active, as seen from the scaling behavior angular dependence of *J*_c_ in Figure 4D, and the *T*_c_ fluctuation caused by those defects becomes the main sources to trap the vortices. On the other hand, elemental non-stoichiometries, as shown in the EPMA mapping in Figure 2, may also contribute to *δT*_c_ pinning. Since the value of *T*_c_ for IBSs is very sensitive to the stoichiometry, those droplets on the film surface will also cause spatial fluctuations of *T*_c_, which contribute to the *δT*_c_ pinning.

In conclusion, we investigated the influence of the superconducting layer thickness on the superconducting properties of Ba122:Co CCs. It was found that the thin film structure quality does not worsen with increasing film thickness, except for small number of impurity. However, pits and droplets appear on the film surface. The influence of thickness on *T*_c_^zero^ weakens with increasing thickness. Dramatic influence on the *J*_c_ by film thickness was observed: below 0.6 μm, the *J*_c_ decreases monotonically with increasing film thickness; the *J*_c_ is almost independent of film thickness in the range of 0.6–1.5 μm; and quick drop in *J*_c_ appears again for film thicker than 1.5 μm. Anisotropic Ginzburg–Landau scaling to the angular dependence *J*_c_ indicates that the 0.2-μm-thick film behaves drastically differently from other films, where correlated pinning centers play the main role in the 0.2 μm and uncorrelated pinning centers play a significant role in the thicker films. However, correlated pinning centers also become active at high field in the vicinity of *H*//*ab*. Transformation from *δl* pinning to *δT*_c_ pinning by thickness variation was discussed in the frame of single vortex pinning regime. However, currently, the *I*_c_ values at high field are still low compared with well-developed PIT tapes of IBSs and YBCO CCs. Therefore, strategies for pinning enhancement should be developed, such as adding artificial pinning centers by secondary phases or irradiations, or reducing the droplet effect by improving target quality.

### Limitations of the study

Although the thickness dependence of the Ba122:Co CCs was investigated for the first time in this work, a large decrease in transport performance with thickness was also observed, suggesting that additional artificial pinning centers, such as defects by irradiation, secondary phase particles, should be introduced to further improve the performance of thick films in the future.

## STAR★Methods

### Key resources table

**Table:** 

REAGENT or RESOURCE	SOURCE	IDENTIFIER
**Software and algorithms**		

Origin 2020	Originlab	http://www.originlab.com/

**Other**		

JEOL JXA-iSP100 Electron Probe Microanalyzer	JEOL (BEIJING) CO., LTD.	http://www.jeol.com.cn/product/detail/556
Bruker-D8 Advance X-ray diffractometer	Bruker (Beijing) Scientific Technology Co. Ltd.	https://www.bruker.com/zh/products-and-solutions/diffractometers-and-scattering-systems/x-ray-diffractometers/d8-advance-family/d8-advance.html
Physical Property Measurement System	Quantum Design China(Beijing)	https://www.qd-china.com/zh/pro/detail3/1/1912091480804/1909260926498

### Resource availability

#### Lead contact

Further information and requests for resources should be directed to and will be fulfilled by the lead contact, Prof. Yanwei Ma (ywma@mail.iee.ac.cn).

#### Materials availability

This study did not generate new unique reagents.

### Method details

Ba122:Co thin films on IBAD-LaMnO_3_ (LMO) buffered metal tapes were fabricated by pulsed laser deposition (PLD) using a KrF excimer laser (wavelength: 248 nm). The target of nominal composition BaFe_1.84_Co_0.16_As_2_ was prepared by the solid-state reaction method (Yuan et al., 2017). The optimal fabrication conditions to obtain high-quality epitaxial thin films were a 1.4∼2 J/cm^2^ laser energy density with a repetition rate of 10 Hz, a 43 mm distance between the target and the substrate and a substrate temperature of 825 °C in a base pressure better than 10^-7^ Torr. After deposition, the films were cooled down to room temperature at a rate of 10 °C/min. We used commercially available metal tapes for YBCO CCs as substrates (courtesy of from Shanghai Creative Superconductor Technologies Co. Ltd). The fabrication details of the buffer architecture, Hastelloy substrate (C-276)/Al_2_O_3_ (80 nm)/Y_2_O_3_ (10 nm)/ IBAD-MgO (10 nm)/MgO (60 nm)/LMO (30 nm), are described in reference (Fan et al., 2020). The LMO buffer layer has a good in-plane lattice matching with YBCO and a better chemical stability. The film thickness was determined by measuring the thickness of the step-edge with a profilometer, where the step was formed by a metal mask during deposition. In addition, different thickness was controlled by deposition time. The thicknesses of as-prepared thin films were varying from 0.2∼2 μm. Crystal structure and phase purity were measured by X-ray diffraction (XRD) with a Cu Kα radiation on a Bruker D8 Advance. The surface topographies and elemental mapping of the Ba122:Co thin films were observed by Electron Probe Microanalyzer (EPMA) (JXA-iSP100, JEOL). The transport critical current (*I*_c_) and the temperature dependence of resistivity of the films were measured with the four-probe method by physical property measurement system (PPMS; Quantum Design) equipped with a sample rotator. The angle of the applied field *θ* was varied from 60° to 240°, where *θ* = 90 and 180° correspond to the configurations of *H//ab*-plane of the films and *H//c*-axis, respectively. Before the measurements of the transport critical current, microbridges of 20 μm in width and 100 μm in length were fabricated by conventional photolithography and Ar^+^ etching. The critical current densities *J*_c_s were calculated from current–voltage (*I–V*) curves with a criterion of 1 μV/cm (with distance between contacts for the voltage readings is about 2-3 mm). The magnetic field *H* was applied in maximum Lorentz force configuration during all measurements.

## Data Availability

This study did not generate any unique data set.
