# Supplementary material for: Thickness dependence of structural and superconducting properties of Co-doped BaFe2As2 coated conductors
Source: iScience. 2021 Jul 30;24(8):102922. doi: 10.1016/j.isci.2021.102922 (PMC8361216; doi:10.1016/j.isci.2021.102922)
Supplement: Document S1. Figures S1 and S2 [file mmc1.pdf]

iScience, Volume *24*

Supplemental information

Thickness dependence of structural and superconducting properties of Co-doped $BaFe_2As_2$ coated conductors

Zhongtang Xu, Chiheng Dong, Chuanbing Cai, Pusheng Yuan, and Yanwei Ma

Supplemental information

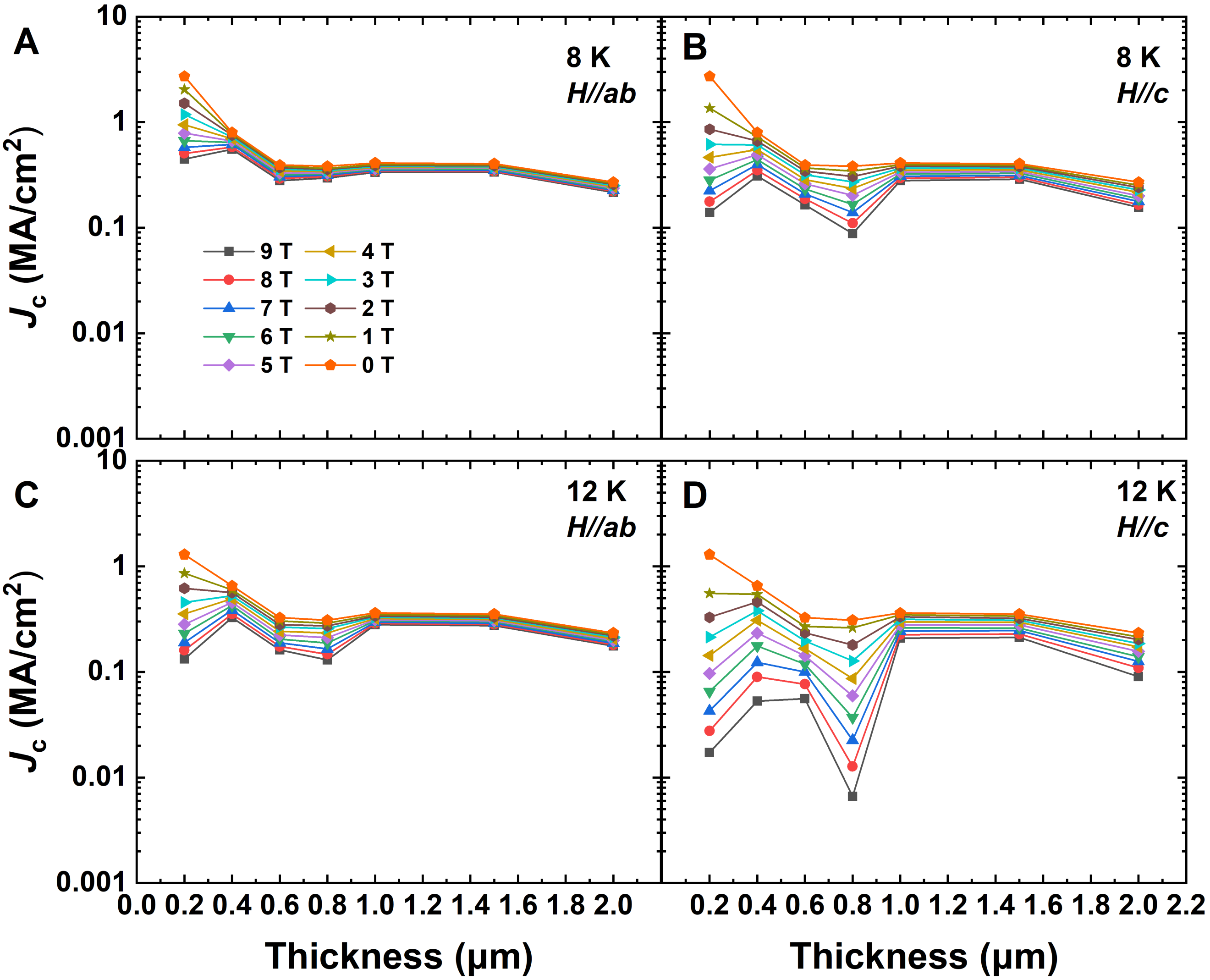

Figure S1. Thickness dependence of J_c

Related to Figure 3. Thickness dependence of J_cs up to 9T for *H//ab* and *H//c* at 8 K and 12 K.

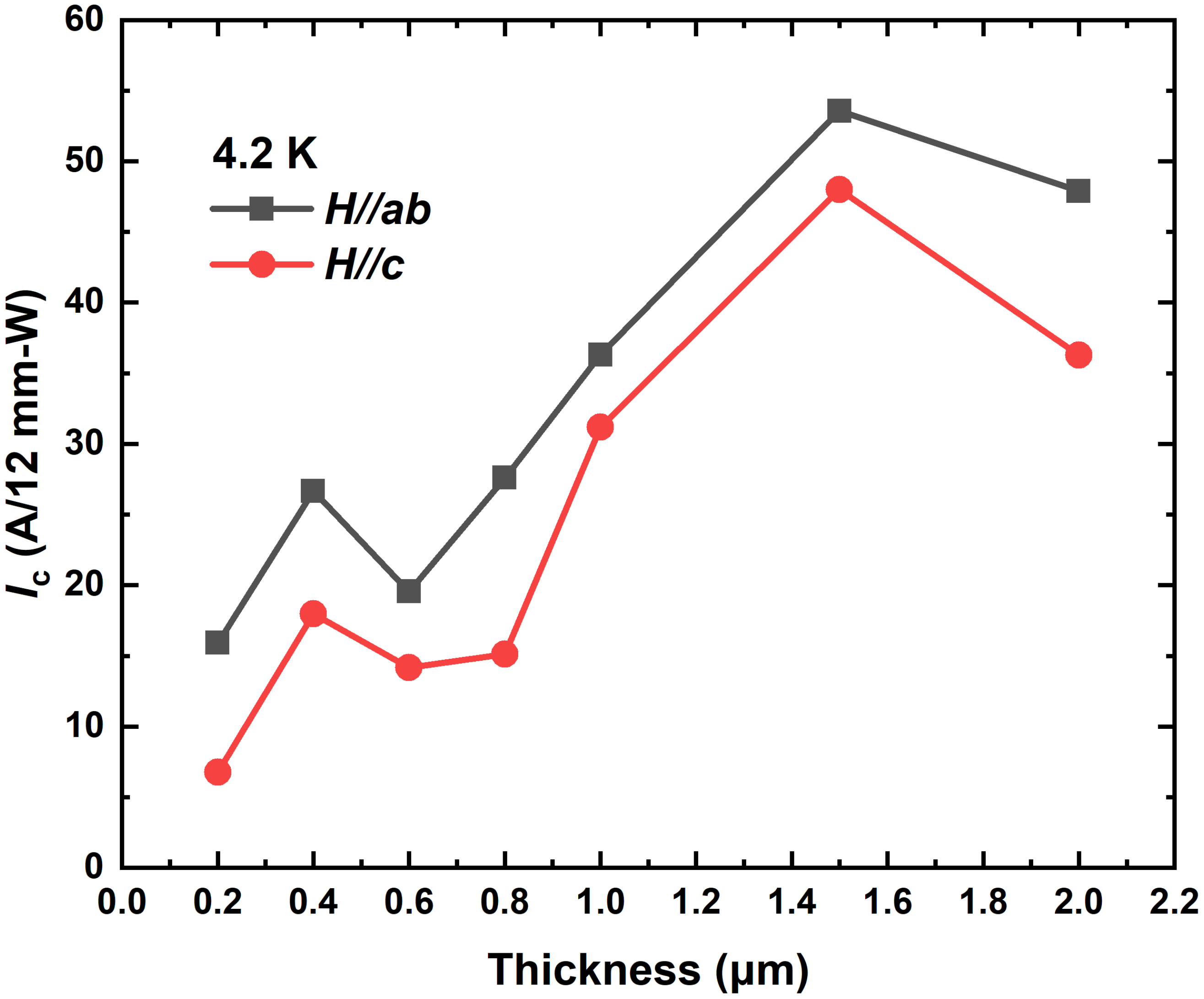

Figure S2. Thickness dependence of critical current I_c

Related to Figure 3. Thickness dependence of crucial current I_c at 4.2 K for for *H//ab* and *H//c* at 9T.
